# Efficacy and safety of generic pomalidomide plus low-dose dexamethasone in relapsed or refractory multiple myeloma: a multicenter, open-label, single-arm trial

**DOI:** 10.1007/s00277-023-05558-y

**Published:** 2023-12-19

**Authors:** Huixing Zhou, Yafei Wang, Jiao Chen, Aili He, Jie Jin, Quanyi Lu, Ying Zhao, Junjun Li, Ming Hou, Liping Su, Xun Lai, Wei Wang, Lihong Liu, Yanping Ma, Da Gao, Wenhong Lai, Xin Zhou, Hongmei Jing, Jinqiao Zhang, Wei Yang, Xuehong Ran, Congmeng Lin, Jianping Hao, Taiwu Xiao, Zhenqian Huang, Zhigang Zhu, Qing Wang, Baijun Fang, Binghua Wang, Yanping Song, Zhen Cai, Bo Liu, Yanan Zhu, Xinai Yang, Xiaoyan Kang, Juan Li, Wenming Chen

**Affiliations:** 1 Department of Hematology, Beijing Chao-Yang Hospital, Capital Medical University, #8, the South Road of Workers Stadium of Chaoyang District, Beijing, 100020 China; 2 Department of Hematology, Tianjin Medical University Cancer Institute and Hospital, Tianjin, China; 3 Department of Hematology, Sichuan Academy of Medical Sciences, Sichuan Provincial People’s Hospital, Chengdu, China; 4 Department of Hematology, The Second Affiliated Hospital of Xi’an Jiaotong University, Xi’an, China; 5 Department of Hematology, The First Affiliated Hospital Zhejiang University School of Medicine, Hangzhou, China; 6 Department of Hematology, Zhongshan Hospital Affiliated to Xiamen University, Xiamen, China; 7 Department of Hematology, The First People’s Hospital of Foshan, Guangzhou, China; 8 Department of Hematology, The First Affiliated Hospital of University of South China, Hengyang, China; 9 Department of Hematology, Qilu Hospital of Shandong University, Jinan, China; 10 Department of Hematology, Shanxi Provincial Cancer Hospital, Taiyuan, China; 11 Department of Hematology, Yunnan Cancer Hospital, The Third Affiliated Hospital of Kunming Medical University, Yunnan Cancer Center, Kunming, China; 12 Department of Hematology, The Affiliated Hospital of Qingdao University, Qingdao, China; 13 Department of Hematology, The Fourth Hospital of Hebei Medical University, Shijiazhuang, China; 14 Department of Hematology, Second Hospital of Shanxi Medical University, Taiyuan, China; 15 Department of Hematology, The Affiliated Hospital of Inner Mongolia Medical University, Hohhot, China; 16 Department of Hematology, The First Affiliated Hospital of Gannan Medical University, Ganzhou, China; 17 Department of Hematology, Wuxi People’s Hospital, Wuxi, China; 18 Department of Hematology, Peking University Third Hospital, Beijing, China; 19 Department of Hematology, The Third Hospital of Hebei Medical University, Shijiazhuang, China; 20 Department of Hematology, Shengjing Hospital of China Medical University, Shenyang, China; 21 Department of Hematology, Weifang People’s Hospital, Weifang, China; 22 Department of Hematology, Zhangzhou Municicap Hospital of Fujian Province, Zhangzhou, China; 23 Department of Hematology, The First Affiliated Hospital of Xinjiang Medical University, Urumchi, China; 24 Department of Hematology, Liaocheng People’s Hospital, Liaocheng, China; 25 Department of Hematology, The First Affiliated Hospital of Guangzhou Medical University, Guangzhou, China; 26 Department of Geriatric Hematologic Oncology, Guangzhou First People’s Hospital, Guangzhou, China; 27 Department of Hematopathology, Guizhou Provincial People’s Hospital, Guiyang, China; 28 Department of Hematology, Henan Cancer Hospital, Affiliated Cancer Hospital of Zhenghzou University, Zhengzhou, China; 29 Department of Hemolymph, Weihai Central Hospital, Weihai, China; 30 Department of Hematology, Xi’an Central Hospital, Xi’an, China; 31 Bone Marrow Transplantation Center, the First Affiliated Hospital of Zhejiang University School of Medicine, Hangzhou, China; 32 Clinical Research Center, Qilu Pharmaceutical Co., Ltd., Jinan, China; 33 Department of Hematology, First Affiliated Hospital of Sun Yat-Sen University, #58, The 2nd Zhongshan Road, Yuexiu District, Guangzhou, 510062 China

**Keywords:** Pomalidomide, Generic drug, Relapse and/or refractory multiple myeloma (RRMM), Dexamethasone

## Abstract

This multicenter, open-label, single-arm trial (ClinicalTrials.gov, NCT05236621) was conducted to confirm the efficacy and safety of generic pomalidomide plus dexamethasone in Chinese patients with relapsed or refractory multiple myeloma (RRMM). Total 79 eligible RRMM patients were planned to be included. Patients were treated with generic pomalidomide (4 mg daily on days 1–21, orally) and low-dose dexamethasone (40 mg/day on days 1, 8, 15, and 22, orally; 20 mg for patients aged > 75 years) in 28-day cycles until disease progression with a maximum treatment duration of 2 years. The primary endpoint is the overall response rate (ORR) assessed by the independent review committee per the 2016 International Myeloma Working Group guidelines. A total of 85 eligible patients were included in this study from 32 centers in China, with a median age of 62.0 (range, 39–76) years, a median prior line of therapy of 4 (range, 1–16), and 41.2% patients with high-risk cytogenetics. The ORR was 38.8% (95% confidence interval (CI), 28.44–50.01). The disease control rate was 67.1% (95% CI, 56.02–76.87), meanwhile, the median progression-free survival was 5.55 months (95% CI, 3.68–7.52). Among the treatment-related adverse events (TRAEs), infective pneumonia (17.6%) was the most frequent non-hematologic adverse event, while a decrease in neutrophil count (52.9%) was the most common grade ≥ 3 TRAE. The study results indicated that the generic pomalidomide demonstrated consistent efficacy and a safety profile similar to the branded pomalidomide when combined with low-dose dexamethasone in Chinese RRMM patients.

**Registration number** ClinicalTrials.gov NCT05236621, retrospectively registered on February 11, 2022.

**Supplementary Information:**

The online version contains supplementary material available at 10.1007/s00277-023-05558-y.

## Introduction

Multiple myeloma (MM) is the second most frequent hematological malignancy, with a median age of 69 years at diagnosis [1]. The standardized prevalence and incidence were 5.68 per 100,000 population and 1.60 per 100,000 person-years, respectively, from 2012 to 2016 in China [2]. In 2022, there were 22,450 new cases of MM and an estimated 17,360 MM-related deaths in China, compared to 33,463 new cases and an estimated 14,145 deaths in the USA, indicating a higher mortality rate in China [3]. MM patients who relapse or refractory to primary therapy and/or maintain therapy with proteasome inhibitors, such as bortezomib, or immunomodulatory drugs (IMiDs), such as lenalidomide, experience a shorter median overall survival (OS) of 3 months or 9 months without or with further treatment, respectively [4]. POM + LD-Dex treatment exhibited more than a partial response in 31%–33% of patients, with a median progression-free survival (PFS) of 4.0–4.2 months and a median OS of 12.7–16.5 months in patients with relapsed and refractory multiple myeloma (RRMM) as observed in the MM-002 and MM-003 trials [5, 6]. Furthermore, when combined with novel specific antibodies targeting CD38, signaling lymphocytic activation molecule (SLAM) family member 7 (SLAMF7), or dual-targeting CD3 and B cell maturation antigen (BCMA), (POM + LD-Dex)-based regimens demonstrated significant beneficial effects in the treatment of RRMM, resulting in a substantially increased overall response rate (ORR) of 53%–69% in RRMM patients [7–12]. Currently, pomalidomide, in combination with dexamethasone with or without an anti-CD38 antibody, is extensively used in the treatment of multiple myeloma for RRMM patients after 1–3 prior therapies, according to the National Comprehensive Cancer Network (NCCN) guidelines [5, 6, 13]. Therefore, pomalidomide plays a pivotal role in the treatment of RRMM. However, the branded pomalidomide has yet to be approved by the National Medical Products Administration (NMPA) of China, as the new drug application was submitted in June 2022. Thus, there is an urgent need for pomalidomide treatment of in China. The generic pomalidomide has been developed, demonstrating its bioequivalence in healthy Chinese subjects (Supplemental file 1). The availability of generic pomalidomide will address the pressing need for RRMM treatment in China. Considering the guidelines for regulatory approval of generic drugs in China, the application must be supported by clinical trial data. This open-label, single-arm trial was conducted to assess the efficacy and safety of generic pomalidomide in Chinese RRMM patients in accordance with the relevant guidelines established by the NMPA in China.

## Material and methods

### Study design and participants

The study drug, pomalidomide, manufactured by Qilu Pharmaceutical Co. Ltd., is a generic version of pomalidomide (Imnovid®, Celgene Corporation, New Jersey, USA). A single-arm, bridging, confirmatory study for generic pomalidomide was recommended, designed in accordance with the MM-003 phase III study [5]. This multicenter, open-label, single-arm study was conducted at 32 centers in China.

The inclusion criteria were as follows: patients aged at least 18 years with an Eastern Cooperative Oncology Group (ECOG) performance status score of 0–2; documented diagnosis of relapsed and refractory multiple myeloma (RRMM) according to the guideline for the diagnosis and management of multiple myeloma in China (2020 revision); having received at least two previous therapies, including two cycles of lenalidomide and two cycles of proteasome inhibitors (bortezomib or ixazomib), with disease progression during prior treatment or within 60 days after completing prior treatment; and having measurable disease defined as a serum monoclonal protein concentration of at least 5 g/L, a urine monoclonal protein of at least 200 mg/24 h, or serum-free light chain of at least 100 mg/L; along with adequate hematological, hepatic, and renal function (absolute neutrophil count (ANC) ≥ 1.0 × 109/L, platelet count ≥ 50 × 109/L, total bilirubin ≤ 2.0 mg/dL, alanine aminotransferase (ALT) and aspartate aminotransferase (AST) ≤ 3 upper limits of normal (ULN), serum creatinine ≤ 3.0 mg/dL, or creatinine clearance of ≥ 30 ml/min as per the Cockcroft-Gault formula [14].

The exclusion criteria were as follows: patients intolerant to thalidomide, lenalidomide, pomalidomide, or hypersensitive to proteasome inhibitors, dexamethasone, or other ingredients; patients with peripheral neuropathy of grade 3 or significant cardiac disease (New York Heart Association class II or above, myocardial infarction within 12 months before enrollment, or unstable or poorly controlled angina pectoris).

All patients provided written informed consent before enrollment in the study. The study protocol received approval from the institutional review board and independent ethics committees of all participating institutions before the initiation of any study procedures. The study was conducted following the Declaration of Helsinki (revised in 2013) and the principles of Good Clinical Practice.

### Procedures

All enrolled patients were administered pomalidomide at a dose of 4 mg on days 1–21 of a 28-day cycle, along with low-dose dexamethasone at 40 mg or 20 mg (for patients aged at least 75 years) on days 1, 8, 15, and 22 of a 28-day cycle. Thromboprophylaxis with low-dose aspirin, low-molecular-weight heparin, or warfarin was strongly recommended for patients at risk of thromboembolism. Laboratory parameters, vital signs, electrocardiograms, ECOG performance status, and imaging were examined every cycle.

During treatment, adverse events (AEs) such as arterial or venous thromboembolism, hematological toxicity, peripheral neuropathy, and pneumonia, among others, were closely monitored. Dose interruptions and reductions were permitted in accordance with the MM-003 phase III study [5]. Per protocol, treatment with pomalidomide was halted when grade 3–4 toxicity occurred and continued with a reduced dose of 3 mg daily until the toxicity recovered to grade 2. Upon a second recurrence of grade 3–4 toxicity, the dose of pomalidomide was to be reduced by 1 mg, and so forth.

After the study was discontinued, patients were followed up to collect information on the date of progression, subsequent treatment for RRMM, and survival every 12 weeks for up to 2 years following the enrollment of the last patient, or at the decision of the sponsor to conclude the study.

Efficacy assessments were conducted in the full analysis set (FAS) population. Efficacy was evaluated on day 1 of every cycle by an independent review committee (IRC) according to the International Myeloma Working Group (IMWG) response criteria [15]. Treatment continued in patients with controlled disease, assessed as stringent complete response (sCR), complete response (CR), very good partial response (VGPR), partial response (PR), minimal response (MR), or stable disease (SD), until progressive disease (PD) or unacceptable toxicity.

The safety analysis was conducted in patients who received at least one dose of the trial treatment and was utilized for all safety analyses. Safety was assessed by evaluating the incidence of adverse events (AEs) in terms of type, frequency, severity, and their relationship to the study drugs. AEs were classified according to MedDRA version 25.0 and graded based on the National Cancer Institute Common Terminology Criteria for Adverse Events (CTCAEs), version 5.0.

Clinical laboratory tests were performed either at a central laboratory or at the laboratories of each research site. Patient information, laboratory test results, and efficacy assessments were collected and recorded in an electronic data collection system. Data analysis and statistical analysis were conducted by an independent data analysis company. The authors take responsibility for all aspects of the work, ensuring that any questions related to the accuracy or integrity of any part of the study are appropriately investigated and resolved.

### Statistical analysis

The primary endpoint was ORR assessed by an independent review committee (IRC), defined as the proportion of patients evaluated as having partial response (PR) or better, including the rates of sCR, CR, VGPR, and PR. The secondary endpoints included duration of response (DoR), disease control rate (DCR), progression-free survival (PFS), overall survival (OS), and safety.

The efficacy of pomalidomide was explored in RRMM patients with different risk-stratification stages of the international staging system (ISS) or revised international staging system (R-ISS) [16].

A sample size of 66 patients was determined using PASS 2019, providing 80% power to detect the difference between the null hypothesis proportion ${\pi }_{0}$ of 0.12 and the alternative proportion ${\pi }_{1}$ of 0.25 at a one-sided significance level of 0.025. Considering a dropout rate of 20%, the sample size was expanded to 79.

The two-sided 95% exact confidence intervals (CI) were calculated using Clopper-Pearson exact method.

The Kaplan–Meier method was employed to estimate time-to-event endpoints and corresponding 95% CIs, including DoR, PFS, and OS. Patient demographics and clinical data were analyzed descriptively. P-values were calculated using Fisher’s Exact Test. Statistical analyses were performed using SAS, version 9.4 or higher.

## Results

### Clinical characteristics of patients

Between March 29, 2021 and November 9, 2022, a total of 109 patents were screened, and finally, 85 eligible RRMM patients were enrolled in the study, all of whom were included in the intent to treat (ITT) population. As of the data cutoff date (December 1, 2022), 65 (76.5%) patients discontinued treatment, with 41 (48.2%) PD, 8 (9.4%) withdrawal of informed consent, 6 (7.1%) for treatment-emergent adverse events (TEAEs), 8 (9.4%) for investigator’s discretion, 1 due to death, and 1 due to major violation of the protocol. A total of 43 (50.6%) patients were still ongoing the study. All 85 patients were included in the ITT, received at least one dose of pomalidomide and one efficacy assessment, and were included in the FAS population.

In the FAS population, the median age was 62.0 (range, 39–76) years, with 34.1% of the patients aged 65 years or older. The majority of patients had an Eastern Cooperative Oncology Group (ECOG) performance status of 0 -1, and 60.0% were male (Table 1). All patients were refractory to both lenalidomide and bortezomib, with 71.8% of patients received cyclophosphamide. Additionally, 9 (10.6%) patients had a treatment history of anti-CD38 antibodies, with 8 patients receiving daratumumab and 1 patient receiving isatuximab. Fourteen (16.5%) patients had prior autologous hematopoietic stem cell transplantation (ASCT). The median time from initial diagnosis to enrollment was 36.3 months (range, 8.5–154.6) for all patients. The median number of previous treatments was 4.0 (range, 1–16); 32 (37.6%) patients had ISS stage III, while 21 (25.6%) patients had R-ISS stage III. High-risk cytogenetics was observed in 35 (41.2%) patients. The most common cytogenetic abnormalities were gain(1q21) (67.1%) and del(13q) (51.8%) (Table 1).

**Table 1:** Baseline disease characteristics (intent-to-treat population)

Baseline Characteristic	Pomalidomide + Low-dose dexamethasone (*N* = 85)
Patients	
Age, year, median (range)	62.0 (39–76)
< 65, n (%)	56 (65.9)
≥ 65 to ≤ 75, n (%)	27 (31.8)
> 75, n (%)	2 (2.4)
Sex, n (%)	
Male	51 (60.0)
Female	34 (40.0)
ECOG performance status, n (%)	
0 or 1	75 (88.3)
2	10 (11.8)
International Staging System stage at study entry, n (%)	
I	30 (35.3)
II	23 (27.1)
III	32 (37.6)
Revised International Staging System stage at study entry^*^, n (%)	
I	12 (14.6%)
II	49 (59.8%)
III	21 (25.6%)
Disease	
Time from initial diagnosis of multiple myeloma to enrollment, median (range), months	36.3 (8.5–154.6)
β2-Microglibulin, median (range), mg/L	3.8 (1.7–15.6)
Cytogenetic profile by FISH, n (%)	
High risk^#^	35 (41.2)
Standard risk	47 (55.3)
Missing data	3 (3.5)
Cytogenetic abnormities, n (%)	
Gain (1q21)	57 (67.1)
Del(13q)	44 (51.8)
del(17p)	18 (21.2)
t(4;14)	19 (22.4)
t(14;16)	0
Multiple myeloma subtype at study entry, n (%)	
lgG	36 (42.4)
lgD	8 (9.4)
lgA	23 (27.1)
Light chain	18 (21.2)
Prior treatments	
Number of prior treatments, median (range)	4.0 (1–16)
Prior treatments in > 50% patients, n (%)	
Lenalidomide	85 (100.0)
Bortezomib	85 (100.0)
Dexamethasone	83 (97.6)
Cyclophosphamide	61 (71.8)
Prior treatment with anti-CD38 antibody, n (%)	
Yes	9 (10.6)
Prior ASCT, n (%)	
Yes	14 (16.5%)

Abbreviations: *FISH* fluorescence in situ hybridization; *ECOG* Eastern Cooperative Oncology Group; *ISS* international stage system; *R-ISS* revised international stage system; *ASCT* autologous hematopoietic stem cell transplantation. ^*^A total of 82 patients were staged per R-ISS for 3 patients were not available with missing cytogenetic FISH results. ^#^High-risk cytogenetic status is defined as the presence of at least one of del (17p13.1), translocation t(4;14), and translocation t(14;16)

### Dosage and modification of pomalidomide and dexamethasone

During treatment, 42 (49.4%) patients had at least one dose adjustment of pomalidomide, with 34 (40.0%) patients experiencing a dose adjustment and 18 (21.2%) patients encountering delayed medication. Twenty-four (28.2%) patients underwent a dose reduction of pomalidomide (24 patients taking a reduced dose of 3 mg daily, 4 patients taking 2 mg daily, and 1 patient taking 1 mg daily). Concerning dexamethasone, 34 (40.0%) patients had at least one adjustment, with 22 (25.9%) patients undergoing a dose adjustment and 20 (23.5%) patients experiencing delayed medication. Eleven patients had their dexamethasone dose reduced to 20 mg.

**Fig. 1:**
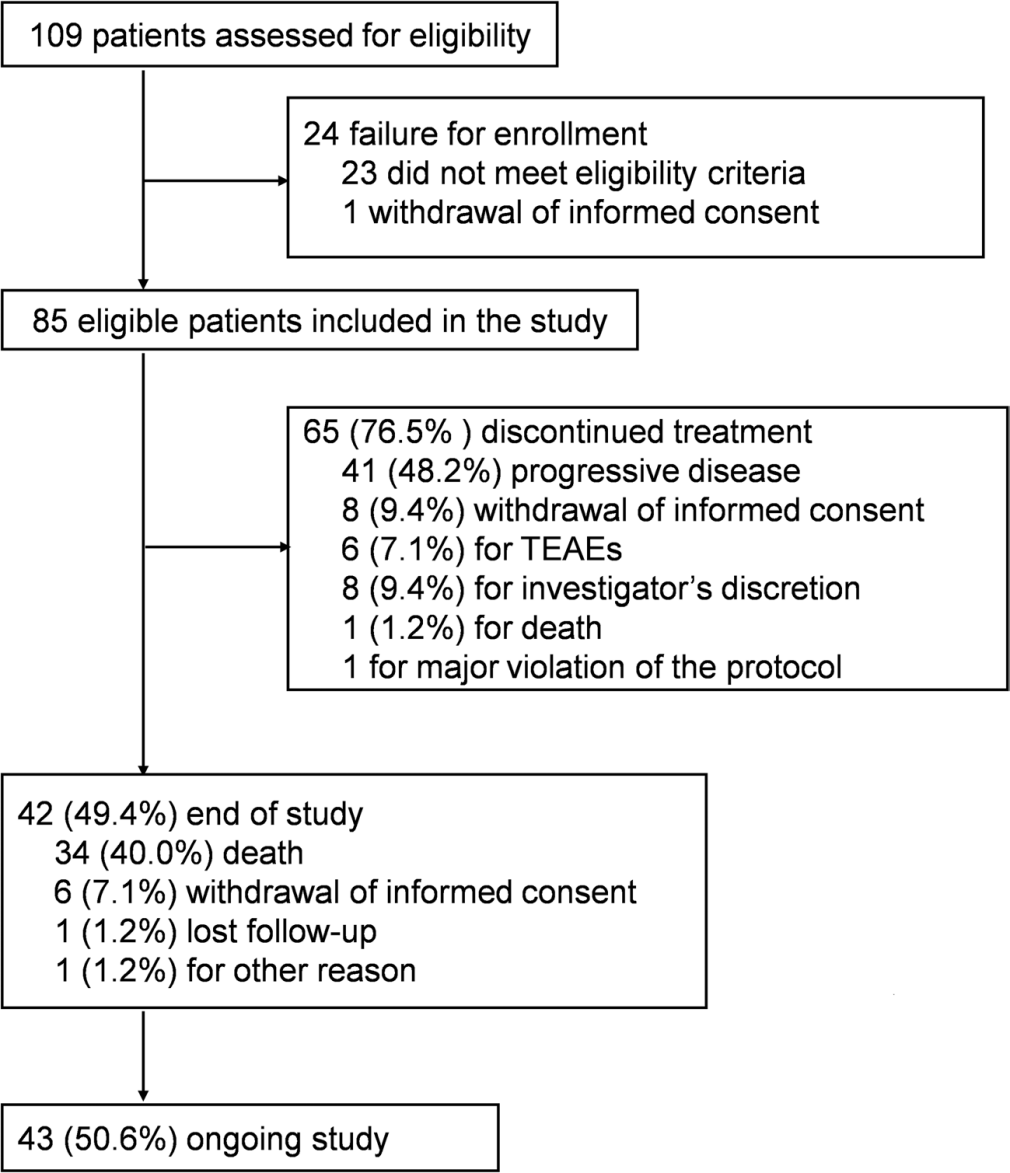
Patient disposition. Abbreviations: TEAEs, treatment emergent adverse events

As of the data cutoff date, 65 (76.5%) patients had discontinued treatment, while 43 (50.6%) patients were still ongoing in the study. Disease progression was the most common reason for discontinuation, observed in 41 (48.2%) patients. Five (5.9%) patients discontinued due to treatment-emerged adverse events (Fig. 1). The median duration of treatment with pomalidomide and dexamethasone was 5.82 months (95% CI, 3.98–7.16) in the FAS population.

### Efficacy of generic pomalidomide

The disease control rate (DCR) was 67.1% (95% CI, 56.02–76.87), with 9 (10.6%) minimal responses (MR) and 15 (17.6%) cases of stable disease (SD) (Supplemental Table 1). A total of 15 (17.6%) patients experienced disease progression. Additionally, 10 (11.8%) patients were evaluated without definitive assessments, while 2 patients did not undergo post-baseline assessments. The efficacy assessed by the investigator was consistent with the IRC's findings (Supplemental Table 1).

**Fig. 2:**
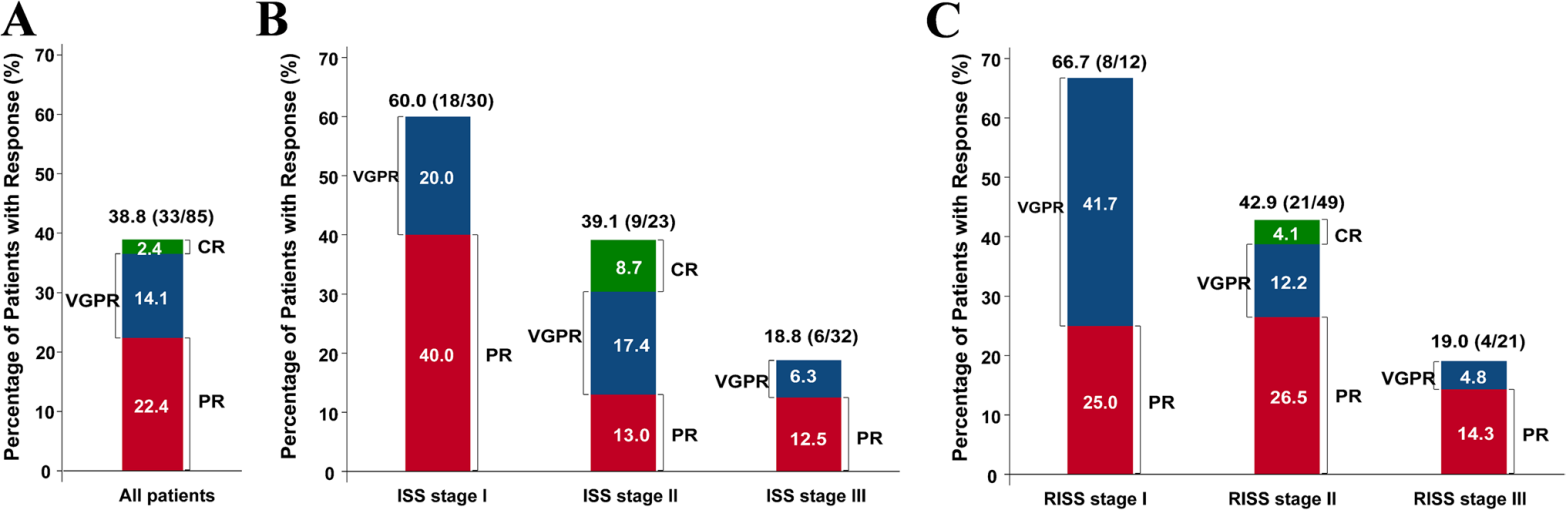
IRC-assessed response to treatment in overall patients (**A**) and in subgroups according to international staging system (ISS) (**B**) and revised international staging system (R-ISS) (**C**). ORRs are based on the number of overall patients. Response was assessed by IRC according to IMWG criteria. Abbreviations: ORR, objective response rate; IRC, independent review committee; IMWG, the International Myeloma Working Group; PR, partial response; VGPR, very good partial response; ISS, international staging system

As of December 1, the ORR was 38.8% (95% CI, 28.44–50.01), including 2 (2.4%) CR, 12 (14.1%) VGPR, and 19 (22.4%) PR according to the IRC assessment in the FAS population (Fig. 2A). The ORR was higher than the predefined ORR of 25%, and the lower 95% CI limit of ORR was higher than the null hypothesis of 12%, indicating that this study successfully achieved its primary endpoint. The DCR was 67.1% (95% CI, 56.02–76.87), with 9 (10.6%) MR and 15 (17.6%) SD (Supplemental Table 1). A total of 15 (17.6%) patients experienced disease progression. Additionally, 10 (11.8%) patients were evaluated without definitive assessments, while 2 patients did not undergo post-baseline assessments. The efficacy assessed by the investigator was consistent with the IRC's findings (Supplemental Table 1).

**Fig. 3:**
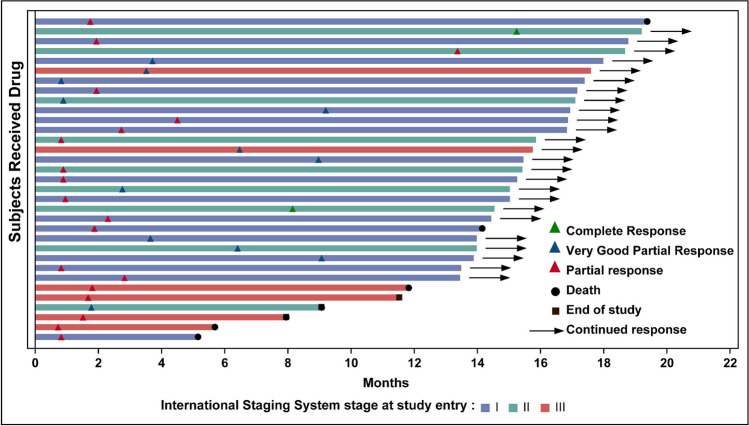
Treatment response in responders (A). Treatment response over time in 32 patients achieved partial response or better

At the time of the data cutoff, among the 33 patients who had at least PR, 14 (42.4%) patients experienced disease progression, 3 (9.1%) patients received further treatment with new anti-tumor therapy, and 16 (48.5%) patients remained under follow-up. The estimated median DoR was 12.98 months (95% CI, 8.74–not reached), with 3-month and 6-month DoR rates of 93.6% (95% CI, 76.6–98.4) and 77.2% (95% CI, 58.0–88.4), respectively (Fig. 3).

**Fig. 4:**
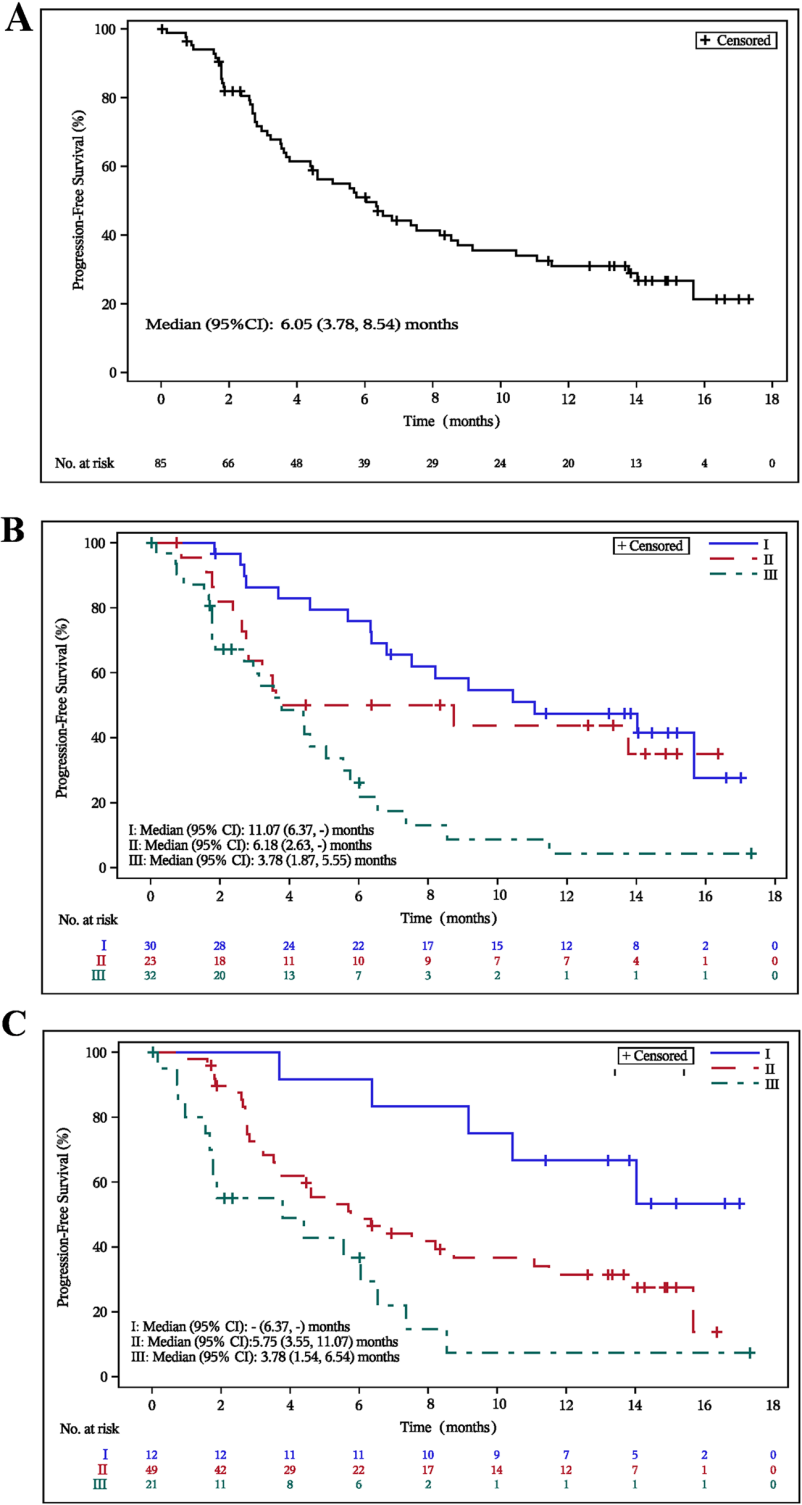
Kaplan-Meier curves of progression-free survival (PFS). The Kaplan-Meier curves of PFS in overall patients (**A**), in subgroups per international staging system (ISS) (**B**) or revised- international staging system (R-ISS) (**C**) in full analysis set

With a median follow-up of 13.24 months (95% CI, 10.81–14.42), 34 (40.0%) patients had died, 6 (7.1%) patients were censored due to withdrawal of informed consent, and 43 (50.6%) were still under survival follow-up. The estimated PFS was 6.05 months (95% CI, 3.78–8.54), with 53 (62.4%) cases of disease progression, 3 (3.5%) deaths, and 18 (21.2%) patients under follow-up. The 6-month and 12-month PFS rates were 51.0% (95% CI, 39.5–61.4) and 31.0% (95% CI, 20.8–41.7), respectively (Fig. 4A).

**Fig. 5:**
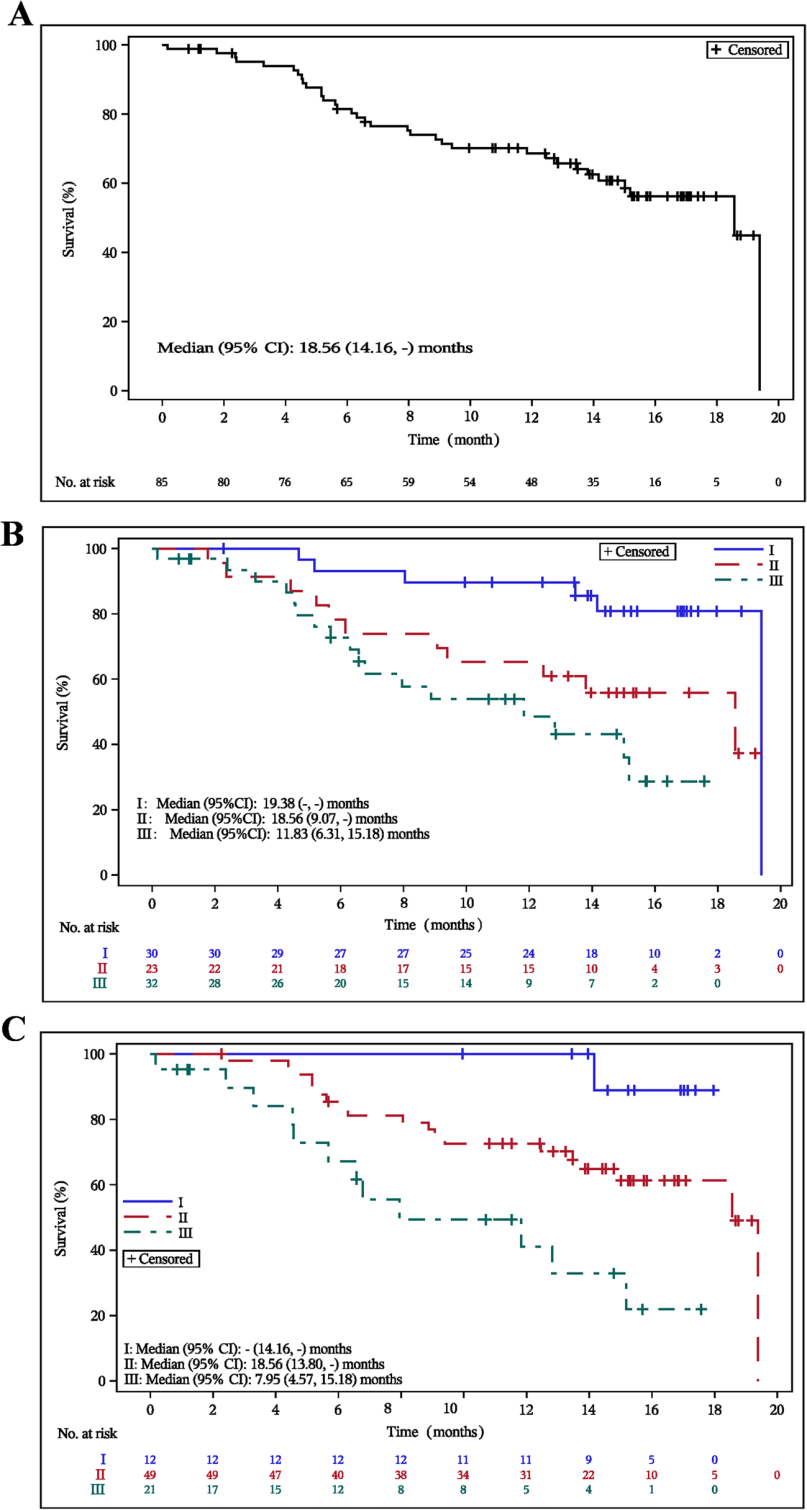
Kaplan-Meier curves of overall survival (OS) in overall patients and subgroups. The Kaplan-Meier curves of OS in overall patients (**A**), in subgroups per international staging system (ISS) (**B**) or revised- international staging system (R-ISS) (**C**) in full analysis set

The estimated median overall survival (OS) was 18.56 months (95% CI, 14.16–not reached). The 6-month and 12-month OS rates were 81.5% (95% CI, 71.3–88.4) and 68.4% (95% CI, 57.3–77.7), respectively (Fig. 5A). After disease progression, 24 patients received subsequent treatment with anti-CD38 antibodies, 3 with selinexor, a selective inhibitor of nuclear export (SINE), 4 with anti-BCMA chimeric antigen receptor T-cell (CAR-T) therapy, and 2 with talquetamab in other clinical trials, a bispecific G-protein-coupled receptor family C group 5 member D (GPRC5D)-directed CD3 T-cell engager.

### Post hoc efficacy analysis in subgroups per ISS and R-ISS

In the post hoc analysis, the ORR and DCR in patients with ISS stage I were both significantly higher compared to those in patients with ISS stage III (ORR: 60.0% (95%CI, 40.6–77.3) vs 18.8% (7.2–36.4), *P* = 0.0015; DCR, 86.7% (69.3–96.2) vs 53.1% (34.7–70.9), *P* = 0.0057) (Fig. 2B, Supplemental Table 2). Concerning R-ISS, the ORR and DCR in patients with stage I were also significantly higher than those in patients with stage III (ORR: 66.7% (95%CI, 34.9–90.1) vs 19.0% (95%CI, 5.5–41.9), *P* = 0.010; DCR, 86.7% (69.3–96.2) vs 47.6% (25.7–70.2), *P* = 0.022) (Fig. 2C, Supplemental Table 2). Notably, patients with cytogenetic standard risk or prior anti-CD38 antibody treatment exhibited similar ORR and DCR compared to those with cytogenetic high risk or naïve anti-CD38 treatment, respectively (Supplemental Table 2).

In the ISS subgroup analysis, the median PFS for patients with stages I, II, and III were 11.07 (95% CI, 6.37–not reached) months, 6.18 (95% CI, 2.63–not reached) months, and 3.78 (95% CI,1.87–5.55) months, respectively (Fig. 4B). The median PFS for ISS stage I and II were significantly longer compared to ISS stage III, with P values of < 0.0001 (stage I vs stage III) and 0.0056 (stage II vs stage III). Median OS for patients with stages I, II, and III were 19.38 (95% CI, not reached–not reached) months, 18.56 (95% CI, 9.07–not reached) months, and 11.83 (95% CI, 6.31–15.18) months, respectively (Fig. 5B). The median OS for RRMM patients with ISS stage I was significantly longer than that for ISS stage III (*P* = 0.0003).

In the R-ISS subgroup analysis, the median PFS for patients with stages I, II, and III were not reached (95% CI, 6.37–not reached) months, 5.75 (95% CI, 3.55–11.07) months, and 3.78 (95% CI,1.54–6.54) months, respectively (See Fig. 4C). The median PFS for R-ISS stage I was significantly longer than that for R-ISS stage III (*P* = 0.0002). Median OS for patients with R-ISS stage I, II, and III were not reached (95% CI, 14.16–not reached) months, 18.56 (95% CI, 13.80–not reached) months, and 7.95 (95% CI, 4.57–15.18) months, respectively. The median OS for R-ISS stage I and II were significantly longer than that for R-ISS stage III, with P values of 0.0003 (stage I vs stage III) and 0.0304 (stage II vs stage III).

### Poste-hoc efficacy analysis in patients exposed to anti-CD38 monoclonal antibodies

In post-hoc analysis, in patients treated with anti-CD38 monoclonal antibodies, the ORR and DCR were 33.3% (95% CI, 7.5–70.1) and 66.7% (95%CI, 30.0–92.5), respectively, with 1 (11.1%) VGPR and 2 (22.2%) PR. In 76 (89.4%) patients previously untreated with anti-CD38 antibody, the ORR and DCR were 38.2% (95% CI, 27.3–50.0) and 65.8% (95% CI, 54.0–76.3), with 7 (9.2%) VGPR and 22 (28.9%) PR. Prior anti-CD38 monoclonal antibody treatment did not make significant difference in response to subsequent pomalidomide. The ORRs were found to be similar in patients with or without prior ASCT, at 35.4% and 39.4%, respectively.

In patients with high-risk cytogenetics, the ORR and DCR were 34.3% (95% CI, 19.13–52.21) and 68.6% (95% CI, 50.71–83.15), respectively, with 1 (2.9%) CR, 3 (8.6%) VGPR, 8 (22.9%) PR, 6 (17.1%) MR, and 6 (17.1%) SD. In patients with standard-risk cytogenetics, the ORR and DCR were 44.7% (95% CI, 30.17–59.88) and 68.1% (95% CI, 52.88–80.91), respectively, with 1 (2.9%) CR, 9 (19.1%)VGPR, 11 (23.4%)PR, 3 (6.4%) MR, and 8 (17.0%) SD. The ORR in patients with high-risk cytogenetics was numerically less than the ORR in patients with standard-risk cytogenetics.

### Safety

All the 85 patients were included in the safety set (SS). A total of 84 (98.8%) patients had at least one TEAEs, with 81 (95.3%) of these cases being attributed to pomalidomide. Additionally, 70 (82.4%) patients had grade ≥ 3 TEAEs, of which 62 (72.9%) were related with pomalidomide. Furthermore, 41 (48.2%) patients underwent treatment-emergent serious adverse events (TESAEs), with 30 (35.3%) cases being related with pomalidomide. Dose adjustment caused by pomalidomide-related TEAEs occurred in 40 (47.1%) patients. Treatment discontinuation for pomalidomide-related TEAEs occurred in 27 (31.8%) patients (Table 2). Following the initiation of POM + LD-Dex treatment, one patient died due to serious adverse events (SAEs) associated with acute myocardial ischemia and heart failure, deemed unrelated with the study treatment and more connected to underlying health conditions. Additionally, one patient died due to systemic organ failure, and two other patients died of disease progression within the initial 2 months after treatment initiation.

**Table 2:** Treatment-emergent adverse events (safety population)

Treatment emergent adverse events (TEAEs)	Pomalidomide + Low-dose dexamethasone (*N* = 85)
Any grade TEAEs	84 (98.8%)
Grade ≥ 3 TEAEs	70 (82.4%)
Pomalidomide-related TEAEs	81 (95.3%)
Pomalidomide-related Grade ≥ 3TEAEs	62 (72.9%)
Treatment-emergent serious adverse events (TESAEs)	41 (48.2%)
Pomalidomide-related TESAEs	30 (35.3%)
TEAEs leading to dose adjustment	42 (49.4%)
Pomalidomide-related TEAEs leading to dose adjustment	40 (47.1%)
TEAEs leading to discontinuation of study drug	29 (34.1%)
Pomalidomide-related TEAEs leading to discontinuation of study drug	27 (31.8%)
AEs leading to discontinuation of study	6 (7.1%)
Pomalidomide-related TEAEs leading to discontinuation of study	6 (7.1%)
Death	4 (4.7%)
Death related with pomalidomide	0

Abbreviations: *TEAEs* treatment-emergent adverse events. TEAEs were named per MedDRA 25.0 and graded per the Common Terminology Criteria for Adverse Events (CTCAE) v5.0

The most prevalent pomalidomide treatment-related adverse events (TRAEs) of any grade included neutrophil count decreased (69.4%), white blood cell count decreased (65.9%), platelet count decreased (50.6%), anemia (34.1%), and lymphocyte count decreased (34.1%). These TRAEs also represented the most frequent grade ≥ 3 TRAEs. (Refer to Table 3) Considering infection as a significant cause of AEs, the incidence of pneumonia and upper respiratory tract infection related to pomalidomide treatment were 11.8% and 5.9%, respectively. The occurrence of TRAEs of interest was relatively low, including 3 (3.5%) cases of peripheral neuropathy, 2 (2.4%) instances of venous thrombosis in the limb, 2 (2.4%) cases of thrombosis, 1 (1.2%) case of deep vein thrombosis, and 1 (1.2%) case of pulmonary embolism.

**Table 3:** Pomalidomide treatment-related adverse events (TRAEs) (safety population)

Preferred terms	Pomalidomide + Dexamethasone (*N* = 85)	
	All grades TRAEs (≥ 5% in frequency)	Grade ≥ 3 TRAEs
Neutrophil count decreased	59 (69.4%)	44 (51.8%)
White blood cell count decreased	56 (65.9%)	27 (31.8%)
Platelet count decreased	43 (50.6%)	16 (18.8%)
Anemia	29 (34.1%)	17 (20.0%)
Lymphocyte count decreased	29 (34.1%)	12 (14.1%)
Pneumonia	10 (11.8%)	9 (10.6%)
Fatigue	10 (11.8%)	2 (2.4%)
Pruritus	10 (11.8%)	0
Astriction	9 (10.6%)	0
Hyperuricemia	8 (9.4%)	0
Hypokalemia	8 (9.4%)	1 (1.2%)
Pneumonitis	6 (7.1%)	3 (3.5%)
Hypoproteinemia	5 (5.9%)	1 (1.2%)
Upper respiratory tract infection	5 (5.9%)	0
Fever	5 (5.9%)	0
Blood bilirubin increased	5 (5.9%)	0

Abbreviations: *TRAEs* treatment-emergent adverse events. TRAEs were named per MedDRA 25.0 and graded per the Common Terminology Criteria for Adverse Events (CTCAE) v5.0

## Discussion

In this open-label, single-arm, multicenter clinical trial, generic pomalidomide demonstrated a similar efficacy and safety profile in Chinese patients with RRMM. In exploratory analysis, RRMM patients with either ISS stage I or R-ISS stage I derived the greatest benefit from POM + LD-Dex treatment to a similar extent. Those with high-risk cytogenetic status exhibited a numerically lower ORR compared to patients with standard risk. Regardless of prior treatment with anti-CD38 antibody, RRMM patients seemed to benefit from pomalidomide treatment.

The study was designed in accordance with the MM-002 and MM-003 trials, implementing intermittent pomalidomide (4 mg, 21/28 days for a cycle) with LD-Dex. The efficacy of POM + LD-Dex in this study was similar to that observed in MM-002 and MM-003 [5, 6, 17]. The synergistic effect of LD-Dex with intermittent pomalidomide resulted in notable tumor regression. Intermittent pomalidomide aided in circumventing acquired resistance to drug-induced immune activation, while the combination with low-dose dexamethasone expedited the activation of innate and adaptive immunity [17].

This study enrolled patients who had received at least 2 prior therapies and exhibited similar characteristics to participants in the MM-002 and MM-003 studies [5, 6]. Notably, this study included patients with prior treatment involving anti-CD38 antibodies, suggesting that these patients could also benefit from POM + LD-Dex treatment.

In this study, the efficacy of POM + LD-Dex appeared to be distinguished by a superior median progression-free survival (PFS) (6.05 vs. 4.0 months) and median overall survival (OS) (18.56 vs. 13.1 months) when compared with the MM-003 study [18]. This discrepancy might be attributed to several factors. Firstly, the efficacy of POM + LD-Dex in the MM-003 study could have been compromised as patients were permitted to switch from the high-dose dexamethasone group to the pomalidomide plus LD-Dex group [5, 18]. Furthermore, in this study, RRMM patients demonstrated the potential for novel subsequent therapies, including anti-CD38 antibodies, SINE, anti-BCMA-CAR-T, and bispecific antibodies targeting GPRC5D and CD3. These emerging therapies contributed to prolonging overall survival compared with the data from the MM-003 study. The median PFS in this study was comparable to the median PFS (6.05 vs. 6.47 months) observed in the POM + LD-Dex group in the ICARIA-MM trial [8]. Moreover, the results of the ORR and median PFS, as assessed by the IRC and the investigator, were consistent, highlighting the robustness of this trial.

In this study, the efficacy of POM + LD-Dex was analyzed in patients with different stages of ISS or R-ISS [16]. Subgroup analysis revealed that patients with ISS stage I exhibited a significantly higher ORR than those with ISS stage III. Patients with ISS stage III demonstrated the lowest ORR, consistent with data from other studies [5, 19]. The OS benefit was more pronounced in stratified patients according to R-ISS. Furthermore, the current study demonstrated that patients who had progressed from anti-CD38 antibody treatment could also benefit from POM + LD-Dex, with comparable efficacy to those without prior anti-CD38 treatment.

In the cytogenetic analysis of the MM-003 study, POM + LD-Dex was found to be effective in RRMM patients with del(17p) or t(4;14), showing significantly longer PFS and OS compared to high-dose dexamethasone [18]. In this study, del(17p), t(14,16), and t(4;14) were defined as high-risk cytogenetic abnormalities. We observed that the ORR in RRMM patients with high-risk cytogenetics was numerically lower than the ORR in patients with standard risk (34.3% vs. 44.7%). In the ICARIA-MM trial, both the ORR and median PFS in patients with high-risk cytogenetics were lower compared to those in (POM + LD-Dex)-treated patients with standard-risk cytogenetics (16.7% vs. 42.3% for ORR, and 3.7 vs. 7.4 months for median PFS) [20]. While direct comparison across trials is not feasible, it is evident that RRMM with high-risk cytogenetics does respond to POM + LD-Dex treatment. Thus, POM + LD-Dex appears to be efficacious for Chinese RRMM patients with high-risk cytogenetics and remains a favorable option for general RRMM patients, irrespective of high-risk cytogenetics [21, 22].

However, in other study, gain(1q21) aberrations was identified as a type of high-risk cytogenetic abnormality with prognostic and clinical implications [23]. RRMM patients may be charactered with increased incidence of high-risk cytogenetic gain(1q21), which is correlated with inferior survival [24]. The efficacy of POM + LD-Dex in patients with three or more copies of isolated gain(1q21) (without other chromosomal abnormalities) was found to be inferior [20]. In this study, the incidence of gain(1q21) was about two-thirds; however, the extent to which gain (1q21) affects anti-tumor activity remains unclear and warrants further exploration in future studies.

In this study, the AEs profile was in consistent with the reported safety profile of POM + LD-Dex in RRMM patients from previous studies [5, 6, 19, 22, 25]. The primary grade ≥ 3 hematologic TEAE was neutropenia, while the primary grade ≥ 3 infectious TEAE was pneumonia. Therefore, POM + LD-Dex exhibited a similar safety profile and was well-tolerated in RRMM patients. Additionally, this study demonstrated that POM + LD-Dex caused similar hematologic AEs and infectious diseases but fewer gastrointestinal disorders in Chinese patients, when compared with the safety profile observed in the MM-011 study involving Japanese RRMM patients [25]. Consequently, pomalidomide has manageable safety for RRMM patients as well as for Asian patients.

However, there are limitations to consider in this study. Firstly, as a single-arm study with a relatively small patient population and a short follow-up period, the patient numbers across ISS subgroups and R-ISS subgroups were imbalanced and limited. Furthermore, excessive censoring within specific subgroups may have impacted the efficacy data. Secondly, the comparison of efficacy and safety profiles across trials was conducted despite differences in patient baseline characteristics and disease features. Thirdly, this study prioritized the ORR as the primary endpoint, while PFS and OS were designated as secondary endpoints for confirmatory purposes. Lastly, the post hoc efficacy analysis was conducted within subgroups based on ISS or R-ISS, emphasizing the necessity for confirmation in a larger-scale phase III trial.

In conclusion, the generic pomalidomide demonstrated comparable efficacy and safety in Chinese RRMM patients, aligning with the findings from previous studies involving the branded pomalidomide. This study reaffirmed that RRMM patients with ISS stage I/R-ISS stage I benefited the most from POM + LD-Dex treatment. Moreover, POM + LD-Dex was found to be beneficial for RRMM patients with prior anti-CD38 antibody treatment and those with high-risk cytogenetic status.

### Supplementary Information

Below is the link to the electronic supplementary material.

Supplementary file1 (DOCX 19 KB)

Supplementary file2 (DOCX 26 KB)

## Data Availability

The Qilu Pharmaceutical Co., Ltd. will provide the study protocol and data with publication of this manuscript as required.
