# Supplementary material for: Efficacy and safety of generic pomalidomide plus low-dose dexamethasone in relapsed or refractory multiple myeloma: a multicenter, open-label, single-arm trial
Source: Ann Hematol. 2023 Dec 19;103(3):855–68. doi: 10.1007/s00277-023-05558-y (PMC10866745; doi:10.1007/s00277-023-05558-y)
Supplement: Supplementary file 1 — Supplementary file1 (DOCX 19 KB) [file 277_2023_5558_MOESM1_ESM.docx]

**Supplemental Table 1. Summary of responses according to independent response committee (IRC) and investigator based on IMWG criteria (intention to treat population).**

| Variable | Pomalidomide plus low dose dexamethasone (N=85) | | |
| --- | --- | --- | --- |
|  | Per IRC | Per investigator | |
| Best overall response, n (%) | | | |
| Stringent complete response (sCR) | 0 | | 2 (2.4) |
| Complete response (CR) | 2 (2.4) | | 2 (2.4) |
| Very good partial response (VGPR) | 12 (14.1) | | 12 (14.1) |
| Partial response (PR) | 19 (22.4) | | 18 (21.2) |
| Overall response rate (ORR), n (%, 95% CI) | 33 (38.8%, 28.4–50.0) | | 34 (40.0%, 29.5–51.2) |
| Minimal response (MR) | 9 (10.6) | | 5 (5.9) |
| Stable disease (SD) | 15 (17.6) | | 24 (28.2) |
| Disease control rate (DCR), n (%, 95%CI) | 57 (67.1%, 56.0–76.9) | | 63 (74.1%, 63.5–83.0) |
| Progressive disease (PD) | 15 (17.6) | | 13 (15.3) |
| Not estimable^#^ | 10 (11.8) | | 4 (4.7) |
| Not estimable^$^ | 3 (3.5) | | 5 (5.9) |
| Duration of response (DoR), median (95% CI), months | 12.98 (8.74–NR) | | 12.98 (8.51–NR) |
| Progression free survival (PFS), median (95% CI), months | 6.05 (3.78–8.54) | | 6.11 (3.81–7.56) |
| Overall survival (OS), median (95% CI), months | 18.6 (14.2–NR) | | |
| 6-month OS rate, % (95% CI) | 81.5 (71.3–88.4) | | |
| 12-month OS rate, % (95% CI) | 68.7 (57.3–77.7) | | |

The date of data analysis was December 1, 2022. Abbreviations: IRC, Independent Review Committee; IMWG, International Myeloma; NR, not reached. According to the guideline of IMWG, any response should be confirmed for sequential two times before initiation of other new treatment. ORR = sCR + CR + VGPR + PR; DCR = sCR + CR + VGPR + PR + MR + SD. Not estimable^#^: patients who had efficacy assessment without identifying the best response. Not estimable^$^: patients without efficacy assessment after enrollment. Working Group.

**Supplemental Table 2. Subgroup analysis of response to pomalidomide plus low dose dexamethasone in relapse and/or refractory multiple myeloma according to independent response committee (IRC) and investigator based on IMWG criteria (intention to treat population).**

| Stratification factor | Response per IRC | | | | | Response per investigator | | | |
| --- | --- | --- | --- | --- | --- | --- | --- | --- | --- |
|  | ≥PR | ORR, % (95% CI) | MR+SD | | DCR, % (95% CI) | ≥PR | ORR, % (95% CI) | MR+SD | DCR, % (95% CI) |
| International staging system at study entry, n or %(95% CI) | | | | | | | | | |
| Stage I (n=30) | 18 | 60.0 (40.6–77.3)* | | 8 | 86.7 (69.3–96.2)^$^ | 19 | 63.3 (43.9–80.1) | 9 | 93.3 (77.9–99.2) |
| Stage Ⅱ (n=23) | 9 | 39.1 (19.7–61.5) | | 5 | 60.9 (38.5–80.3) | 9 | 39.1 (19.7–61.5) | 7 | 65.2 (42.7–83.6) |
| Stage Ⅲ (n=32) | 6 | 18.8 (7.2–36.4） | | 11 | 53.1 (34.7–70.9） | 6 | 18.8 (7.2–36.4) | 14 | 62.5 (43.7–78.9) |
| Revised-International staging system at study entry^#^, n or %(95% CI) | | | | | | | | | |
| Stage I (n=12) | 8 | 66.7 (34.9–90.1)^&^ | 3 | | 91.7 (61.5–99.9)^#^ | 10 | 83.3 (51.6–97.9) | 2 | 100.0 (73.5–100.0) |
| Stage II (n=49) | 21 | 42.9 (28.8–57.8) | 14 | | 71.4 (56.7–83.4) | 20 | 40.8 (27.0–55.8) | 19 | 79.6 (65.7–89.8) |
| Stage III (n=21) | 4 | 19.0 (5.5–41.9) | 6 | | 47.6 (25.7–70.2) | 4 | 19.0 (5.5–41.9) | 7 | 52.4 (29.8–74.3) |
| Cytogenetic profile by FISH, n or % (95% CI) | | | | | | | | | |
| High risk (n=35) | 12 | 34.3 (19.1–52.2) | 12 | | 68.6 (50.7–83.2) | 11 | 31.4 (16.9–49.3) | 14 | 71.4 (53.7–85.4) |
| Standard risk (n=47) | 21 | 44.7 (30.2–59.9) | 11 | | 68.1 (52.9–80.9) | 23 | 48.9 (34.1–63.9) | 14 | 78.7 (64.3–89.3) |
| Prior anti-CD38 antibody treatment, n or % (95% CI) | | | | | | | | | |
| Yes (n=9) | 3 | 33.3 (7.5–70.1) | 3 | | 66.7 (29.9–92.5) | 3 | 33.3 (7.5–70.1) | 3 | 66.7 (29.9–92.5) |
| No (n=76) | 30 | 39.5 (28.4–51.4) | 21 | | 67.1 (55.4–77.5) | 31 | 40.8 (29.7–52.7) | 26 | 75.0 (63.7–84.2) |

Abbreviations: ORR, overall response rate; DCR, disease control rate; sCR, stringent complete response; CR, complete response; VGPR, very good partial response; PR, partial response; MR, minimal response; SD, stable disease; IRC, Independent Review Committee; FISH, fluorescence in situ hybridization. ORR = sCR + CR + VGPR + PR; DCR = sCR + CR + VGPR + PR + MR + SD. High-risk cytogenetic status is defined as the presence of at least one of del(17p13.1), translocation t(4;14), and translocation t(14;16). *p=0.0015, compared with ISS stage III; ^$^p=0.0057, compared with ISS stage III; ^&^p=0.010, compared with R-ISS stage III; ^#^p=0.022, compared with R-ISS stage III.
