# Supplementary material for: Efficacy and safety of generic pomalidomide plus low-dose dexamethasone in relapsed or refractory multiple myeloma: a multicenter, open-label, single-arm trial
Source: Ann Hematol. 2023 Dec 19;103(3):855–68. doi: 10.1007/s00277-023-05558-y (PMC10866745; doi:10.1007/s00277-023-05558-y)
Supplement: Supplementary file 2 — Supplementary file2 (DOCX 26 KB) [file 277_2023_5558_MOESM2_ESM.docx]

**Supplemental file 1**

**Bioequivalence of generic pomalidomide capsules in healthy Chinese male subjects**

HUANG Jie1, GU Xing-li2, ZHANG Zuo-kai2, YANG Xiao-yan1, YANG Shuang1, YAO An1, CUI Chang1, YANG Guo-ping1

(1. Center of Clinical Pharmacology, The Third Xiangya Hospital, Central South University, Changsha, China, 410013. 2. Qilu Pharmaceutical Co., Ltd)

**[Summary]**

**OBJECTIVE:** To evaluate the pharmacokinetic characteristics of pomalidomide capsules in healthy Chinese male subjects, and to evaluate the bioequivalence of the generic pomalidomide of 4 mg doses.

**METHODS:** Bioequivalence tests were conducted in fasting and fed state, respectively. A total of 36 healthy male subjects were recruited in each group, in state and fed state, respectively. The generic pomalidomide and pomalidomide capsules (Imnovid^®^) were taken orally. A randomized, open-label, two-cycle, two-crossing fasting state and self-controlled fed state test method was adopted. The plasma drug concentrations were determined at 19 different time points within 48 h by liquid chromatography-tandem mass spectrometry (LC-MS). Main pharmacokinetic parameters, including C_max_, AUC_0-t_ and AUC_0-∞,_ were calculated to assess the bioequivalence.

**RESULTS:** The geometric mean ratios of C_max_, AUC_0-t_ and AUC_0-∞_ were 102.70%, 103.56% and 103.45%, respectively, and the 90% confidence intervals were 98.48%-107.10%, 101.03%-106.16%, and 100.89%-106.07%, respectively, after administration of generic pomalidomide and reference pomalidomide in fasting state, within the range of 80.00% to 125.00%, meeting the criteria for bioequivalence. The geometric mean ratios of main pharmacokinetic parameters C_max_, AUC_0-t_ and AUC_0-∞_ were 101.23%, 102.98% and 103.11%, respectively, and the 90% confidence intervals were 96.80-105.86%, 100.22-105.81% and 100.39-105.91% in the fed state, respectively, within the range of 80.00% to 125.00%, meeting the criteria for bioequivalence.

**Conclusion:** The generic pomalidomide of 4 mg doses showed bioequivalence in healthy Chinese male subjects under both the fasting and fed state.

**[Key words]** Pomalidomide capsules; multiple myeloma; LC-MS/MS; bioequivalence
